# New Summary Measures of Population Health and Well-Being for Implementation by Health Plans and Accountable Care Organizations

**DOI:** 10.5888/pcd13.160224

**Published:** 2016-07-07

**Authors:** Thomas E. Kottke, Jason M. Gallagher, Sachin Rauri, Juliana O. Tillema, Nicolaas P. Pronk, Susan M. Knudson

**Affiliations:** Author Affiliations: Jason M. Gallagher, Sachin Rauri, Nicolaas P. Pronk, Susan M. Knudson, Health Partners, Minneapolis, Minnesota; Juliana O. Tillema, HealthPartners Institute for Education and Research, Bloomington, Minnesota.

## Abstract

Health plans and accountable care organizations measure many indicators of patient health, with standard metrics that track factors such as patient experience and cost. They lack, however, a summary measure of the third leg of the Triple Aim, population health. In response, HealthPartners has developed summary measures that align with the recommendations of the *For the Public’s Health* series of reports from the Institute of Medicine. (The series comprises the following 3 reports: *For the Public’s Health: Investing in a Healthier Future, For the Public’s Health: Revitalizing Law and Policy to Meet New Challenges,* and *For the Public’s Health: The Role of Measurement in Action and Accountability*.) The summary measures comprise 3 components: current health, sustainability of health, and well-being. The measure of current health is disability-adjusted life years (DALYs) calculated from health care claims and death records. The sustainability of health measure comprises member reporting of 6 behaviors associated with health plus a clinical preventive services index that indicates adherence to evidence-based preventive care guidelines. Life satisfaction represents the summary measure of subjective well-being.

HealthPartners will use the summary measures to identify and address conditions and factors that have the greatest impact on the health and well-being of its patients, members, and community. The method could easily be implemented by other institutions and organizations in the United States, helping to address a persistent need in population health measurement for improvement.

**Editor’s Note:** This article is a joint publication initiative between *Preventing Chronic Disease* and the National Academy of Medicine.

## Introduction

In 2008 Berwick, Nolan, and Whittington described pursuit of the Triple Aim as a strategy to improve the US health care system (1), and in 2011 the US Department of Health and Human Services adopted the National Quality Strategy as a driver for better, more affordable care for individuals and the community (2). Also in 2011, the Institute of Medicine (IOM), in a report on the role of measurement in action and accountability, stated that, “Because a summary measure of population health . . . would serve as a marker of the progress of the nation and its communities in improving health, it is important that it be implemented in data collection and public communication efforts at the federal, state, and local levels” (3). If individual health plans were to collect and report a uniform set of summary measures of health and well-being, they would make a significant contribution to implementing the recommendations made in the 2011 report *For the Public’s Health: The Role of Measurement in Action and Accountability*. When aggregated across health plans, the measures would be applicable regionally and nationally, because nearly 90% of Americans are now registered with a health plan (4). In pursuit of efforts to measure progress toward mission achievement, HealthPartners has aligned its efforts with the challenge of the 2011 IOM report on measurement and developed summary measures of health and well-being that can be implemented by any health plan or accountable care organization much in the way that any plan can measure cost of care using HealthPartners’ total cost of care metric (5).

## About HealthPartners

Founded in 1957, HealthPartners is the largest consumer-governed, nonprofit health care organization in the nation. It has a mission of improving health and well-being in partnership with its members, patients, and community. HealthPartners provides a full range of health services including insurance, care delivery, and health and well-being programs. The HealthPartners care system includes a multispecialty group practice of more than 1,700 physicians, 7 hospitals, 52 primary care clinics, 22 urgent care locations, 22 dental clinics, and many specialty practices in Minnesota and western Wisconsin. HealthPartners employs more than 22,500 people, all working together to pursue the HealthPartners mission.

## HealthPartners Summary Measures Framework

It is impossible to create a single measure that comprises both current health and sustainability of health, and health is not the same construct as well-being (6). Therefore, HealthPartners summary measures framework comprises 3 components: a measure of current health, a measure of likelihood of sustainability of health, and a measure of well-being.

### The first component measure: assessing current health

As do the Global Burden of Disease project (7) and the World Health Organization (8), HealthPartners is using disability-adjusted life years (DALYs) as the measure of current health (Box). DALYs comprise years of potential life lost (YLL), which is an estimate of the burden of death on a population, and years lived with disability (YLD), which is an estimate of the burden of nonfatal disease and disability on a population. In a particular year, YLL for the population is calculated by summing, for all individuals who die younger than 75, the difference between age at death and 75. The County Health Rankings project uses a similar measure to report the burden of mortality on a population (9).

**Box. Data Sources for the Summary Measures of Health and Well-Being**

**Table:** 

Framework Element	Source
**Disability-adjusted life years (DALYs)**	
Years of life lost	State death records and administrative data
Years lived with disability	Administrative data
**Sustainability of health**	
Health behaviors	Survey data
Clinical preventive services index	Administrative data
Subjective well-being	Survey data
Social, economic, and physical environment	Administrative and survey data

To define the extent to which a particular disease or disability burdens an individual, the Global Burden of Disease project surveyed approximately 30,000 individuals in Bangladesh, Indonesia, Peru, Tanzania, and the United States. The respondents were presented with pairs of conditions and asked to select the condition that represented the larger disease burden. Correlations between individual survey results and results from analysis of the pooled data set were 0.9 or higher in all surveys except in Bangladesh, where the correlation was 0.75 (10). The exercise resulted in weights for 220 conditions. For example, an untreated spinal cord lesion at the neck has a Global Burden of Disease weight of 0.673 while mild impairment of distant vision has a weight of .004 (10). This implies that an individual with the spinal cord lesion loses about two-thirds of a healthy year because of their condition while the individual with mild impairment of distant vision loses somewhat greater than one day of healthy life. An individual who has no medical, dental, or mental health conditions would have a YLD score of 0.0.

The Global Burden of Disease nomenclature for YLD is not used, however, by HealthPartners and other US health plans. Therefore, HealthPartners created a crosswalk between the weights for the 220 YLD conditions defined by the Global Burden of Disease project and the Johns Hopkins Adjusted Clinical Groups (ACG) system (http://acg.jhsph.org/). The ACG system aggregates medical care claims data into 267 Expanded Diagnosis Clusters, which are in turn aggregated into 27 Major Expanded Diagnosis Clusters. The HealthPartners framework calculates a YLD score for each health plan member using the highest-weight condition in each Major Expanded Diagnosis Cluster using a 100% sample of claims data. As with YLL, YLD is a period prevalence calculation.

### The second component measure: assessing sustainability of health

The sustainability of health measure comprises member reporting of 6 behaviors associated with health plus a clinical preventive services index that indicates adherence to evidence-based preventive care guidelines. The 6 behaviors — tobacco use, fruit and vegetable consumption, physical activity, alcohol use, sleep adequacy, and healthy thinking — were selected because they have a powerful influence on sustainability of health. For example, patterns of tobacco and alcohol use, diet, and physical activity collectively account for approximately 40% of all deaths in the United States (11,12), and a low-risk behavioral pattern has been associated with as many as 10 to 14 years of increased longevity in several populations (13,14). Among HealthPartners’ members who have completed a health assessment as part of an employment-based health promotion program, adherence to recommended behaviors related to fruit and vegetable consumption, physical activity, use of tobacco and alcohol, and adequate levels of sleep is associated with lower incidence of new diagnoses after 2 years (15), better emotional and mental health status (16), lower health care costs, and less productivity loss (17). Importantly, HealthPartners also showed that focused interventions can improve health behaviors (18).

### The third component measure: assessing subjective well-being

Good mental health is more than the absence of mental illness (19), and it is not dependent on the absence of physical disease. The hallmarks of good mental health are “flourishing” and life satisfaction. Flourishing has several related definitions, including “a mindset in which positive affect outweigh(s) negative affect,” (19–21) and “achieving 1 or more of 5 goals: positive emotion, engagement, positive social relations, meaning and purpose, and achievement” (21). In addition to having implicit value for the individual, life satisfaction is associated with outcomes that are important to society. For example, individuals who report high levels of life satisfaction on the HealthPartners health assessments also tend to report higher overall productivity, lower health care and pharmacy costs, and higher adherence to behaviors that promote health. In addition to the positive impact of flourishing on quality of life, individuals who are flourishing have significantly lower risk of death than other individuals, even after adjustment for known risk factors (22).

HealthPartners has chosen life satisfaction as its summary measure of subjective well-being, and in 2015 it began surveying members about life satisfaction and 7 domains that affect subjective well-being: emotional functioning, physical functioning, career satisfaction, adequacy of financial resources, social/interpersonal relations, community support, and meaning and purpose. Its goal is to obtain 5,500 completed surveys per year. “Well-being adjusted life years,” a construct developed for the Institute for Healthcare Improvement’s 100 Million Healthier Lives campaign (23), can be calculated from these data.

## Using the Summary Measures to Promote Health and Well-Being

The measurement of health and well-being will be useful to HealthPartners for planning and assessment throughout the organization. As an aid to clinical services development, the measure can assess the effect of care on years of life lost, increased sustainability of current health through receipt of clinical preventive services, respondent-reported functioning, and subjective well-being.

The summary measures of health and well-being development described in this article offer the potential for generating “top line” population level performance information. For individuals and teams working in the community, the measure has the potential to identify health and well-being disparities. The measure also has the potential to assess the impact of intervention on years of life lost, years lived with disability, sustainability of health, respondent-reported functioning, and objective and subjective well-being. The summary measures of health and well-being can help an organization understand how well its health and well-being improvement agenda is performing, and it can inform evolution of that agenda by providing knowledge about current health status, sustainability of current health, and well-being.

The summary measures of health and well-being could also be used to measure progress toward national initiatives such as the Centers for Disease Control and Prevention’s (CDC’s) 6|18 program (24). The goal of the 6|18 program is to accelerate evidence into action by focusing on 18 goals in 6 areas: tobacco use, high blood pressure, health care–associated infections, asthma, unintended pregnancies, and diabetes. HealthPartners has intervention programs in each of these areas and has the ability to report progress in performance. If all health plans did the same, progress toward these and similar goals could be monitored at local, regional, and national levels.

### Limitations and strengths of the measures

Although the registration of deaths in the United States is complete, the cause of death listed on the death certificate is not always specific, individuals may be misidentified, and the lag time to obtain death records from a state health department or the CDC is 15 months from the end of a particular year. Even with those considerations, however, overall death rates and causes of death can be considered to be adequately reliable.

The estimation of YLD from claims data are potentially biased by the inability of individuals to access care or a low propensity to seek care. Discrepancies between self-reported health and estimates of the burden of disease based on claims data, if they exist, suggest that patients are having difficulty accessing care.

The depth of information that HealthPartners can collect from its members through surveys is limited; all survey response rates are falling, and the US population is fatigued from the ubiquity of surveys. Despite best efforts, challenges remain in identifying alternative methods for obtaining survey responses that preserve the unique opportunities for understanding the relationships between behavior and disease that are created when survey responses can be linked with data on health care use at the individual level.

Because of its brevity, the survey must be seen only as a screening instrument, with collection of more detailed data needed if deficits or problems are identified. Data collected through surveys can also be subject to response bias, both because of the differential propensity for some individuals or subpopulations to complete surveys and because the manner in which they respond to particular survey items may differ. Although statistical adjustment and careful attention to key variables that confound the results can mitigate these shortcomings, concern about residual bias will always exist. Limiting comparisons to situations that are known to be appropriate will also mitigate shortcomings in the data.

These limitations must be weighed against the fact that action must be taken and policy must be made whether or not data are available. HealthPartners believes that policy is best when it is informed by data, even if those data are not perfect.

## Assessing the Social, Economic, and Physical Environment

HealthPartners will use the County Health Rankings framework to assess the elements in the social, economic, and physical environments that affect health (9). The social and economic factors to be assessed include high school graduation rates and proportions of individuals with some college, unemployment rates, percentage of children who live in poverty, adequacy of social support, children in single-parent households, violent crime rates, and rates of deaths from injury. The physical environment elements to be assessed will include stability and quality of housing, time spent commuting to work and whether commuting alone, air pollution, and drinking water violations.

## Plans for the Future

HealthPartners recognizes especially the value of assessing the health and well-being of infants, children, and adolescents. It also recognizes that the health and well-being measures that apply to these groups are not the same as those that apply to adults; the goal of health and well-being promotion for infants, children, and adolescents is to maximize an upward trajectory of development, while the goal for adults is to maintain functioning. HealthPartners expects to develop summary measures of health and well-being for children and adolescents over the next 3 to 5 years.

HealthPartners also recognizes the importance of assessing the health and well-being of individuals with limited English proficiency. The 4 languages spoken most frequently by the population the organization serves, other than English, are Spanish, Somali, Hmong, and Vietnamese. Starting with Hmong, the survey will be translated, tested, and fielded in 1 additional language each year between 2016 and 2019.

## Summary

HealthPartners has developed summary measures of health and well-being that the organization will use to identify and address the conditions that create the highest burden of disease and greatest impact on the health and well-being of its patients and members. It will also use the measures to guide its community-directed initiatives. The method could be implemented by any US health plan.

In an appendix to *Crossing the Quality Chasm,* Plsek advised the reader who wants to design a health system for the 21st century, “[R]ather than agonizing over plans . . . generate a ‘good enough plan’ and begin to observe what happens” (25). HealthPartners has sought to lead the improvement of care and the promotion of health in the past, and it intends to continue to contribute in the future. Consistent with a key IOM recommendation (3), HealthPartners believes that now is the time to create summary measures of health and well-being and to use them to guide the future of health care and the promotion of health and well-being.

## References

[1] Berwick DM , Nolan TW , Whittington J . The triple aim: care, health, and cost. Health Aff (Millwood) 2008;27(3):759–69. 10.1377/hlthaff.27.3.759 18474969

[2] Agency for Healthcare Research and Quality. About the National Quality Strategy; 2016. http://www.ahrq.gov/workingforquality/about.htm. Accessed April 9, 2016.

[3] Committee on Public Health Strategies to Improve Health, Board on Population Health and Public Health Practice. For the public’s health: the role of measurement in action and accountability. Washington (DC): The National Academies Press; 2011. 24983050

[4] Key facts about the uninsured population. The Henry J. Kaiser Family Foundation; 2015. http://kff.org/uninsured/fact-sheet/key-facts-about-the-uninsured-population/. Accessed April 9, 2016.

[5] HealthPartners. Total cost of care; 2014. https://www.healthpartners.com/tcoc. Accessed November 27, 2014.

[6] Kottke TE , Stiefel M , Pronk NP . “Well-Being in All Policies”: promoting cross-sectoral collaboration to improve people’s lives. Prev Chronic Dis 2016;13:160155. 10.5888/pcd13.160155 27079650 PMC4852755

[7] Murray CJL , Lopez AD . Measuring the global burden of disease. N Engl J Med 2013;369(5):448–57. 10.1056/NEJMra1201534 23902484

[8] World Health Organization. Health statistics and information systems. Metrics: disability-adjusted life year (DALY); 2014. http://www.who.int/healthinfo/global_burden_disease/metrics_daly/en/. Accessed November 27, 2014.

[9] University of Wisconsin Population Health Institute. County health rankings and roadmaps; 2016. http://www.countyhealthrankings.org/our-approach. Accessed February 15, 2016.

[10] Salomon JA , Vos T , Hogan DR , Gagnon M , Naghavi M , Mokdad A , Common values in assessing health outcomes from disease and injury: disability weights measurement study for the Global Burden of Disease Study 2010. Lancet 2012;380(9859):2129–43. 10.1016/S0140-6736(12)61680-8 23245605 PMC10782811

[11] McGinnis JM , Foege WH . Actual causes of death in the United States. JAMA 1993;270(18):2207–12. 10.1001/jama.1993.03510180077038 8411605

[12] Mokdad AH , Marks JS , Stroup DF , Gerberding JL . Actual causes of death in the United States, 2000. JAMA 2004;291(10):1238–45. 10.1001/jama.291.10.1238 15010446

[13] Khaw K-T , Wareham N , Bingham S , Welch A , Luben R , Day N . Combined impact of health behaviours and mortality in men and women: the EPIC-Norfolk prospective population study. PLoS Med 2008;5(1):e12. 10.1371/journal.pmed.0050012 18184033 PMC2174962

[14] Fraser GE , Shavlik DJ . Ten years of life: Is it a matter of choice? Arch Intern Med 2001;161(13):1645–52. 10.1001/archinte.161.13.1645 11434797

[15] Pronk NP , Lowry M , Kottke TE , Austin E , Gallagher J , Katz A . The association between optimal lifestyle adherence and short-term incidence of chronic conditions among employees. Popul Health Manag 2010;13(6):289–95. 10.1089/pop.2009.0075 21090987

[16] Pronk NP , Katz AS , Gallagher J , Austin E , Mullen D , Lowry M , Adherence to optimal lifestyle behaviors is related to emotional health indicators among employees. Popul Health Manag 2011;14(2):59–67. 10.1089/pop.2010.0007 21090986

[17] Pronk NP . An optimal lifestyle metric: four simple behaviors that affect health, cost, and productivity. Health Fitness Journal 2012;16:39–43.

[18] Thygeson NM , Gallagher J , Cross K , Pronk NP . Employee health at BAE Systems: an employer-health plan partnership approach. In: Pronk NP, editor. ACSM’s Worksite Health Handbook: a guide to building healthy and productive companies. 2nd edition. Champaign (IL): Human Kinetics 2009:318-26.

[19] Keyes CL . Promoting and protecting mental health as flourishing: a complementary strategy for improving national mental health. Am Psychol 2007;62(2):95–108. 10.1037/0003-066X.62.2.95 17324035

[20] Fredrickson BL , Losada MF . Positive affect and the complex dynamics of human flourishing. Am Psychol 2005;60(7):678–86. 10.1037/0003-066X.60.7.678 16221001 PMC3126111

[21] Seligman M . Flourish. New York (NY): Simon and Schuster; 2011.

[22] Keyes CL , Simoes EJ . To flourish or not: positive mental health and all-cause mortality. Am J Public Health 2012;102(11):2164–72. 10.2105/AJPH.2012.300918 22994191 PMC3477942

[23] Stiefel MC , Riley CL , Roy B , Ramaswamy R , Stout S . 100 Million Healthier Lives measurement system: progress to date. 100 Million Healthier Lives Metrics Development Team Report. Cambridge (MA): Institute for Healthcare Improvement; 2016.

[24] Hester J , Auerbach J , Seeff L , Wheaton J , Brusuelas K , Singleton C . CDC’s 6|18 Initiative: accelerating evidence into action. Washington (DC): National Academy of Medicine; 2016.

[25] Plsek P . Redesigning Health care with insights from the science of complex adaptive systems. In: Committee on Quality of Health Care in America, editor. Crossing the quality chasm. Washington (DC): National Academies Press; 2001. p. 309-22.

